# Cutaneous adverse events associated with long-term immunomodulating therapy in multiple sclerosis

**DOI:** 10.1186/1479-5876-9-S2-P14

**Published:** 2011-11-23

**Authors:** Deepak MW Balak, Gerald JD Hengstman, Raymond Hupperts, H Bing Thio

**Affiliations:** 1 Dept. of Dermatology, Erasmus Medical Center, Rotterdam, The Netherlands; 2 Dept. of Neurology, Catharina Ziekenhuis, Eindhoven, The Netherlands; 3 Regionaal MS Centrum Oost-Brabant, Eindhoven, The Netherlands; 4 Academic MS Centre Limburg, Orbis Medical Centre, Sittard, The Netherlands

## Introduction

Multiple sclerosis (MS) is a common immune-mediated inflammatory disease of the central nervous system that causes severe neurological disability [1]. Treatment is aimed at reducing disease progression via modulation of the immune system with disease-modifying therapies (DMTs) such as glatiramer acetate (GA) and interferon beta (IFN beta) [2]. Skin reactions to DMT are common and involve localized inflammatory processes [3].

## Aim

Our aim was to assess the prevalence and type of cutaneous adverse events associated with long-term use of DMT.

## Methods

A cross-sectional study was conducted in 2010-2011 among 15 clinics in the Netherlands. Eligible for inclusion were MS patients who were treated with their first DMT for at least 2 years. All consecutive eligible patients willing to participate were enrolled, irrespective of the presence of skin reactions. Skin reactions were assessed from digital photographs of the injection-sites by dermatologists, who were blinded for the DMT.

## Results

A total of 146 patients were enrolled. The median age was 47 years (interquartile range [IQR] 41-54 years) and most patients (76%) were female. The median duration of DMT treatment was 4 years (IQR 3-8). Forty-four (30%) patients were treated with intramuscular (IM) IFN beta-1a, 43 (29%) with subcutaneous (SC) IFN beta-1a, 38 (26%) with IFN beta-1b, and 21 (14%) with GA. The proportion of patients with cutaneous adverse events was 40%, 77%, 63%, and 81% among patients receiving IM IFN beta-1a, SC IFN beta-1a, SC IFN beta-1b, and GA, respectively. Skin reactions were local injection-site reactions (61%), lipoatrophy (24%), healed skin ulcers (7%), postinflammatory hyperpigmentation (4%), urticaria (3%), and skin necrosis (1%).

## Conclusion

The prevalence of cutaneous adverse events associated with DMT treatment was high. The most common skin reactions were local injection-site reactions and lipoatrophy related to panniculitis.
